# Tragal Perichondrium and Composite Cartilage Graft Complimenting Endoscopic Tympanoplasty in True Sense: A Comparison

**DOI:** 10.22038/ijorl.2021.48789.2613

**Published:** 2021-05

**Authors:** Arvind Varma, Chetan Bansal, Virendra-Pratap Singh

**Affiliations:** 1 *Department of ENT, Shri Guru Ram Rai Institute of Medical and Health Science Patel Nagar, Dehradun.*; 2 *Senior Medical Officer ONGC Hospital Ballupur Dehradun.*; 3 *Senior Consultant, ENT, Max Superspeciality Hospital Mussorriee Road, Dehradun.*

**Keywords:** Transcanal endoscopic, tympanoplasty, Tragal

## Abstract

**Introduction:**

In days of scar less surgeries it has become necessary for otologists to concentrate on tympanoplasties without external incisions. This study compares the anatomical and functional results of tragal perichondrium graft and perichondrium-cartilage composite graft for transcanal endoscopic tympanoplasties in Himalayan region.

**Methods and Materials:**

This prospective study included 60 subjects of chronic otitis media (mucosal type), who underwent transcanal endoscopic underlay type I tympanoplasty and were divided into two groups. In 30 cases tragal perichondrium graft and in rest of 30 cases Tragal perichondrium Cartilage composite graft was used. Anatomical and functional outcomes were evaluated at 6 months time.

**Results:**

Hearing gain comparing Audiometric data between the tragal perichondrial graft group and tragal perichondrial cartilage composite graft group at 6 months showed no statistically significant differences (P= 0.9533). Assessment of anatomical outcome indicated a greater number of complications in the tragal perichondrial graft group although it was not statistically significant (P=0.6360 in anterior graft failure group, P =0.1322 in reperforation group and P= 0.1056 in retraction group).

**Conclusion:**

Functional results validated both the grafting material while anatomical results are slightly better in tragal perichondrial cartilage composite graft group in term of re perforation and retraction. Moreover use of tragal grafts endoscopic tympanoplasty fulfils its true meaning as no visible scar and post operative patient morbidity is prevented.

## Introduction

Different grafting materials have been used for repair of perforated tympanic membrane and osscicular chain reconstruction. An ideal grafting material is one which has low rejection rate, adequate tensile strength, and similar vibratory function like tympanic membrane. It is adequate in quantity and available near surgical site. Temporalis fascia remains the graft of choice by most otologists. For harvesting temporalis fascia we need an external incision which not only carries risk of scar but also chances of numbness in the area. Nevertheless, it has been associated with 25 -30% of re-perforations in type 1 tympanoplasty (1,2). Moreover, it is also associated with atelectasis of neomembrane over the period of time. In Buchaingm study (3), 89 cases of perforation were repaired using temporalis fascia graft or perichondrium and out of 63 ears available for follow up period of 10 years, 54 ears developed cholesteatomas and 9 had perforations . Temporalis fascia and Perichondrium have been used for repair of atelectatic tympanic membrane. Looking at the statistics of Buckingham study it is likely that fascia and perichondrium graft cases will carry high chances of atelectasis or reperforation (3). 

Over the last decade, use of cartilage as graft has gained popularity in high risk cases, such as those with chronic tubal dysfunction, atelectatic cases, recurrent perforation, nasal allergies, unhealthy middle ear mucosa, subtotal or total perforation, bilateral perforation and pediatric cases. Cartilage is a proven ideal graft in these conditions because of its mechanical properties providing greater resistance to reperforation and retraction. It has low metabolic rate and good acceptance in middle ear (4,5). Because of the thickness and stiff nature of cartilage graft, concerns have been raised regarding reduced vibratory properties. Many studies have shown that slicing the cartilage may overcome this problem. Two main techniques of cartilage tympanoplasty have been described in the literature .First is cartilage-perichondrium island technique and second is palisade technique (6-8), and in both the techniques cartilage from tragus or cymba is taken (1,2,9). Autologous cartilage, due to its stiffness and convexity , can better withstand negative middle ear pressure and chronic nature of middle ear infections (10). 

The aim of this prospective study was to compare anatomical and functional results of no visible scar endoscopic type1 tympanoplasty in tragal perichondrium graft (group A) and tragal perichondrium cartilagecomposite graft (group B) for a follow up period of six months in high altitude Himalayan region. This study also happens to be first of its kind which compares tragal perichondrium and tragal perichondrium composite graft in single handed endoscopic transcanal type I tympanoplasty technique without any external scar.

## Material and Methods

**Fig 1:**
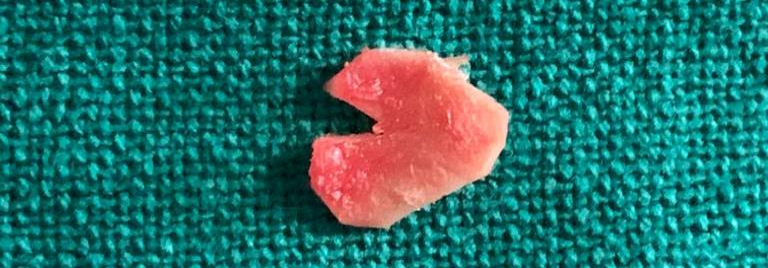
Harvested tragal perichondrium cartilage composite graft

**Fig 2:**
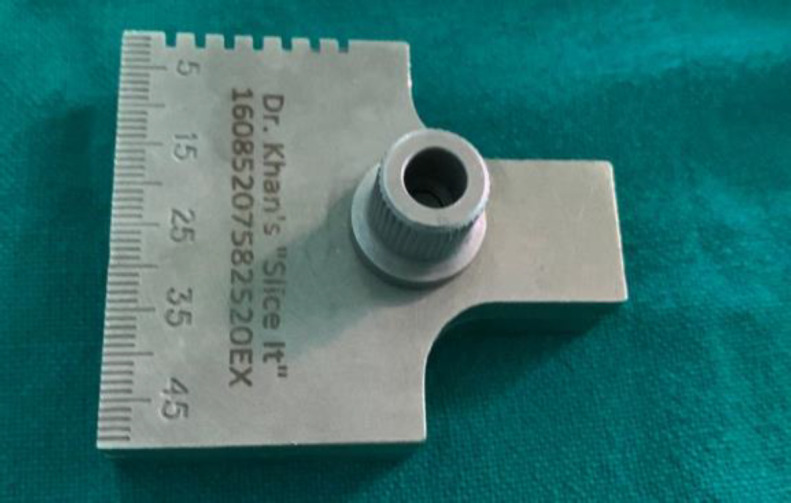
Cartilage slicer used to attain desired thickness of cartilage

tragal perichondrium was used while in group B tragal cartilage along with medial perichondrium was used **(**Figure 1). Cartilage slicer (Figure 2) is used to reduce the thickness of harvested graft to 0.5mm. 

Subjects:

In each group 30 subjects, who underwent single handed transcanal endoscopic type I tympanoplasties between June 2016 to June 2018, were taken for study in a tertiary care centre. To reduce variability in subject sampling and results only subjects undergoing primary tympanoplasties, subjects in age group 10 to 50 yrs were taken to avoid age related sensorineural hearing loss and surgeries performed by single senior surgeon were taken for study. Moreover only unilateral inactive disease with moderate and large perforations was included. In both the groups patients with Eustachian tube dysfunction, patients with adenoids & patients with nasal pathology were excluded from study.Subjects with preoperative and postoperative audiogram with six months regular follow up were only included for study.

Surgical technique:

In all cases transcanal endoscopic type 1 tympanoplasty using perichondrial or perichondrium cartilage graft with underlerlay technique was done under general anesthesia. It was performedusing wide angle 0 degree endoscope. Graft was harvested from tragus (Figure 1) using microscope leaving the apex rim of 2 mm of tragal cartilage and lateral perichondrium to prevent cosmetic deformity.

All subjects underwent procedure in similar steps except the graft material used. In groupA only medial

2% xylocaine and adrenaline infiltration of external auditory canal and tragus using 26 gauze needle was done. A cotton wick containing adrenaline was kept in the external auditory canal .Incision in skin of medial wall of tragus is made 2mm below the tip with surgical blade (No.15) under microscope. A pair of small pointed scissor was use to separate soft tissue from medial surface of tragal cartilage. An incision was made in medial perichondrial layer and it was elevated using round and flap knife in cases of tragal perichondrial graft. In tragal perichondrial-cartilage composite graft lateral perichondrial layer is separated using sharp dissection while medial perichondrium along with cartilage is incised and taken as composite graft. Incision was closed using 4-0 vicryl. Wedge resection of cartilage from the peripheral to central region of the tragal perichondrial graft for placement of malleus handle was performed.

The cartilage graft was sliced using slicer to 0.5 mm thickness. Perforation margins were freshened and tympanosclerotic patches were dissected and removed. 

**Fig 3:**
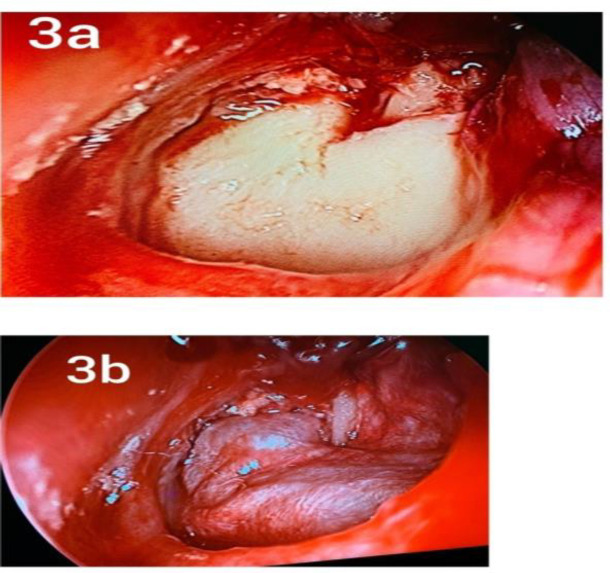
a. Tragal perichondrium cartilage composite graft in left ear after reposition of tympanomeatal flap. b.Tragal perichondrium graft in left ear after reposition of tympanomeatal flap

Incision at osseocartiliginous junction was made and tympanomeatal flap along with annulus was elevated.Entire Middle ear cavity with all the ventilator pathways including anterior and posterior tympanic isthmus was inspected for glue, granulations and disease cleared.Ossicular integrity was checked and round window reflex was elicited. In few cases where handle of malleus was too medialized, handle of malleus was cut using malleus nipper. Graft placed under handle of malleus in perichondrial group and fitted as lock and key in wedge shaped space of tragal perichondrial cartilage graft group (Figure 3 a& b). The middle ear was filled with gel foam. Tympanomeatal flap was reposited back.Gel foam impregnated with topical antibiotic and wick was kept in external auditory canal.Followup was done at 5 days, 1 month, 3 months and 6 months.

Audiology

Pure tone audiometry was done preoperatively and compared to audiogram taken in 6^th^ postoperative month. Pure tone average was calculated using frequencies 500, 1000, 2000 and 4000 Hz. The Air Bone gap was calculated preoperatively and postoperatively and distributed in four groups <10dB HL (decibel Hearing Label) , 11dBHL to 20 dB HL ,21dB HL-30dB HL, >31dB HL.

Otoendoscopic evaluation

**Fig 4:**
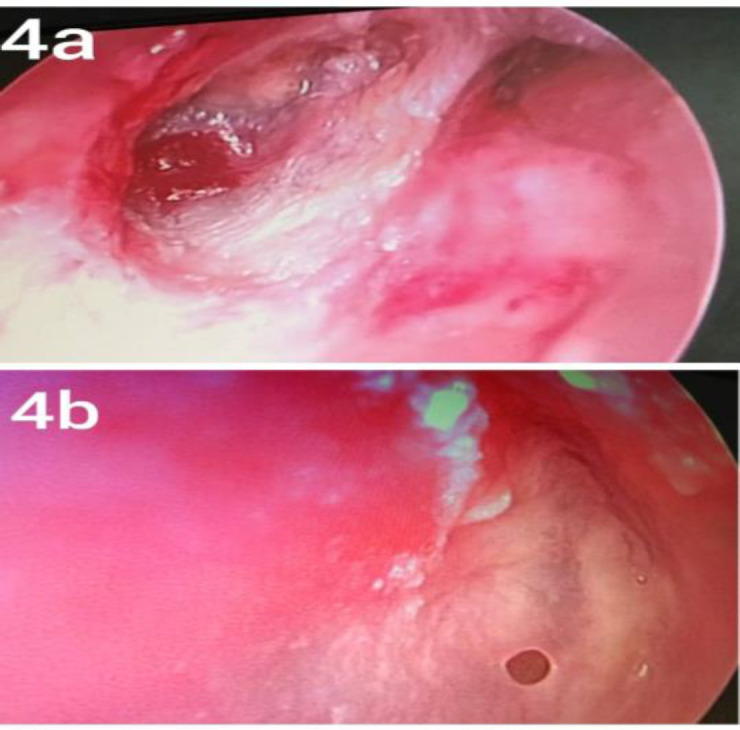
a. Anterior graft failure in anterosuperior quadrant in right ear. b. Reperforation in right ear

All subjects underwent otoendoscopy at the end of 1 month, 3^rd^month and 6^th^ month to inspect neomembrane and especially to see for anterior graft failure (Figure 4a), retraction or reperforation (Figure 4b). Anterior graft failure is an almost invisible gap between anterior edge of graft and anterosuperior tympanic wall lateral to Eustachian tube opening. Retraction was assessed by Valsalva maneuver using Gersdorff staging system for tympanic membrane retraction for pars tensa and Tos staging system for pars flaccida tympanic membrane retraction

Gersdorff staging system for pars tensa tympanic membrane retraction:

Stage I- mobile, can be aspirated by Valsalva or politzer even if adheres to incus

Stage IIa- Fixed, fundus is visible by microscope

Stage IIb- Fixed, fundus is visible by otoendoscope

Stage III- Fixed, fundus is not visible by otoendoscope

Stage IV- Stage III+ accumulation of Keratin debris

Stage V- Cholesteatoma with purulent otorrhea.

Tos staging system for pars flaccida tympanic membrane retraction:

Stage I- pars flaccid is dimpled and is more retracted than normal

Stage II- The retraction pocket is adherent to malleus head

Stage III- The retraction pocket may be hidden.There may be associated erosion of the scutum

Stage IV- There is definite erosion of scutum. The fundus cannot be clearly seen.

Statistical analysis 

At the end of the study, analysis of the groups was done and the results were analyzed statistically, using Chi Square Test and Student t-test, using SSPS III software. P value of less than 0.05 was considered significant and less than 0.001 as highly significant.

## Results

- Functional Evaluation

- Air conduction threshold evaluation(ACT)

In this study postoperativeAir conductionthreshold (ACT) of two groups was significantly better as compared to pre-operative air conduction thresholds (p value < 0.0001) .

In tragal perichondrial graft PG (group A) preoperative mean ACT was 33.3dB HL (SD ± 2.7dB HL) whereas postoperative mean ACT was 20.6 dBHL (SD±4.2dB HL). In tragalperichondrial-cartilage composite PCG (group B) the preoperative mean ACT was 33.3dBHL (SD±2.7dB HL) whereas postoperative mean ACT was 21.2dB HL (SD ±4.4dB HL). As shown in (Table.1).

But, when the postoperative ACT of both the groups was compared it was statistically not significant (P= 0.9533).

**Table 3:** Post-operative oto- endoscopic findings in both groups

**Duration**	**1 month ** **Post-operative**		**3** **month** ** Post-operative**		**6** ** month** ** Post-operative**		**P value using Chi square**
Types of GRAFT	PG (Group A)	PCG (Group B)	PG (Group A)	PCG (Group B)	PG (Group A)	PCG (Group B)	
Anterior Graft failure	3 (10%)	2(6.6%)	3 (10%)	2 (6.6%)	3 (10%)	2 (6.6%)	P = 0.6360
Reperforation	-	-	2 (7.4%)	0 (0%)	2 (7.4%)	0 (0%)	P= 0.1322
Retraction	-	-	-	-	4 (16%)	1 (3.5%)	P= 0.1056

## Discussion

Probably this is the first study which evaluates the anatomical and functional aspects of ideal graft for transcanal endoscopic type I tympanoplasties without any external visible scar. Tragus is the only area available for grafting material in scar less surgeries as scar on medial aspect of tragus is hidden; this provides membranous and cartilaginous graft from the same site and does not need large incision for harvesting. The benefit of this approach goes beyond cosmetics. It also has less chances of infection, less post-operative pain, numbness and shortened recovery time. Moreover, endoscope bypasses the narrow segment of ear canal and provides otologists a wide field view that allows the surgeon to look all around even with 0 degree endoscope which helps in improving results of tympanoplasties.

From functional aspect, in this study hearing thresholds in both the study groups showed an improvement postoperatively. Findings were established statistically by changes in Air conduction threshold (ACT) and Air Bone gap closure (ABG) (p value <.01).Similar results were also observed in other studies De Seta E et al (11), Matthew JG et al (12), Kirazi T et al^7^and Chen XW et al. (13) But when we compared the hearing thresholds post operatively between two grafting material it was statistically not significant (P>.05).

In anatomical aspect, graft uptake rate (successful formation of neotympanum with normal mobility on Valsalva maneuver on otoendoscopy) was 90% in tragal perichondrial graft while it was 94% in tragal perichondrial cartilage composite graft group at the end of one month. The anterior graft failure of 10% in tragal perichondrium group may be due to shrinkage of graft while in tragal perichondrial cartilage group anterior graft failure is 6.6% which may be due to curling of edges of sliced cartilages due to curling of perichondrium on same side. Tos has mentioned four anticurling incisions which may help to solve this. This is statistically insignificant (P>0.05).The results are almost similar to study done by Deseta et al. (11). In this study reperforation was seen in 7.4% cases in tragal perichondrial graft (PG) and no reperforation was seen in tragal perichondrial cartilage composite graft (PCG) at the end of 3 month. The timing of early reperforation in this study may be due to low grade middle ear mucosal infectionwhich was clinically not evident. In addition to anterior graft failure and reperforation at end of 1 month and 3 months retraction was seen in 16% cases in tragal perichondrium graft group (PG) and in 3.5 % in tragal perichondrial cartilage composite graft group (PCG) at the end of 6 months. This is statistically insignificant (P>0.05). This confirms that from anatomical perspective tragal perichondrial cartilage composite graft is more suitable graft material for tympanoplasty in high risk cases such as cases having large perforation, Eustachian dysfunction, in patients with nasal allergy & people residing in high altitude. Harvesting adequate tragal cartilage perichondrium and composite graft needs expertise and has learning curve without compromising cosmesis. We would like to advocate use of operating microscope for magnified view and for adequate, precise harvesting of grafting material.

The strength of present study was selection of subjects and skill of single surgeon to reduce subject variability and results. This made present study more academically beneficial, more homogeneous and easy to compare.

## Conclusion

This study concluded that both the grafting material for transcanal endoscopic type I tympanoplasty give similar functional results but from anatomical perspective tragal perichondrial cartilage composite graft is superior for no scar tympanoplasties. This study lacked long term follow up and had limited number of participants. It demands studies with long term follow up and similar grafting material for better comparison. If similar results are expected to be seen in large studies with long term follow up then tragal perichondrial cartilage graft can be the preferred choice for transcanal endoscopic type I tympanoplasties with no scar. More over with use of tragal grafts endoscopic tympanoplasty fulfils its true meaning as no visible scar and post operative patient morbidity is prevented.
